# Neighborhood Deprivation and Breast Cancer Mortality Among Black and White Women

**DOI:** 10.1001/jamanetworkopen.2024.16499

**Published:** 2024-06-12

**Authors:** Lauren E. Barber, Maret L. Maliniak, Leah Moubadder, Dayna A. Johnson, Jasmine M. Miller-Kleinhenz, Jeffrey M. Switchenko, Kevin C. Ward, Lauren E. McCullough

**Affiliations:** 1 Department of Epidemiology, Emory University Rollins School of Public Health, Atlanta, Georgia; 2 Department of Population Health Science, University of Mississippi Medical Center, Jackson; 3 Department of Biostatistics and Bioinformatics, Emory University Rollins School of Public Health, Atlanta, Georgia

## Abstract

**Importance:**

Neighborhood deprivation has been associated with increased breast cancer mortality among White women, but findings are inconsistent among Black women, who experience different neighborhood contexts. Accounting for interactions among neighborhood deprivation, race, and other neighborhood characteristics may enhance understanding of the association.

**Objective:**

To investigate whether neighborhood deprivation is associated with breast cancer mortality among Black and White women and whether interactions with rurality, residential mobility, and racial composition, which are markers of access, social cohesion, and segregation, respectively, modify the association.

**Design, Setting, and Participants:**

This population-based cohort study used Georgia Cancer Registry (GCR) data on women with breast cancer diagnosed in 2010 to 2017 and followed-up until December 31, 2022. Data were analyzed between January 2023 and October 2023. The study included non-Hispanic Black and White women with invasive early-stage (I-IIIA) breast cancer diagnosed between 2010 and 2017 and identified through the GCR.

**Exposures:**

The Neighborhood Deprivation Index (NDI), assessed in quintiles, was derived through principal component analysis of 2011 to 2015 block group–level American Community Survey (ACS) data. Rurality, neighborhood residential mobility, and racial composition were measured using Georgia Public Health Department or ACS data.

**Main Outcomes and Measures:**

The primary outcome was breast cancer–specific mortality identified by the GCR through linkage to the Georgia vital statistics registry and National Death Index. Cox proportional hazards regression was used to estimate age-adjusted and multivariable-adjusted hazard ratios (HRs) and 95% CIs for the association between neighborhood deprivation and breast cancer mortality.

**Results:**

Among the 36 795 patients with breast cancer (mean [SD] age at diagnosis, 60.3 [13.1] years), 11 044 (30.0%) were non-Hispanic Black, and 25 751 (70.0%) were non-Hispanic White. During follow-up, 2942 breast cancer deaths occurred (1214 [41.3%] non-Hispanic Black women; 1728 [58.7%] non-Hispanic White women). NDI was associated with an increase in breast cancer mortality (quintile 5 vs 1, HR, 1.36; 95% CI, 1.19-1.55) in Cox proportional hazards models. The association was present only among non-Hispanic White women (quintile 5 vs 1, HR, 1.47; 95% CI, 1.21-1.79). Similar race-specific patterns were observed in jointly stratified analyses, such that NDI was associated with increased breast cancer mortality among non-Hispanic White women, but not non-Hispanic Black women, irrespective of the additional neighborhood characteristics considered.

**Conclusions and Relevance:**

In this cohort study, neighborhood deprivation was associated with increased breast cancer mortality among non-Hispanic White women. Neighborhood racial composition, residential mobility, and rurality did not explain the lack of association among non-Hispanic Black women, suggesting that factors beyond those explored here may contribute to breast cancer mortality in this racial group.

## Key Points

**Question:**

Is neighborhood deprivation associated with breast cancer mortality among Black and White women and do interactions with race and neighborhood characteristics representing access, social cohesion, and segregation modify the association?

**Findings:**

In this cohort study of 36 795 non-Hispanic Black and White patients with breast cancer, neighborhood deprivation was associated with increased breast cancer mortality among non-Hispanic White women, but not among non-Hispanic Black women, regardless of the modifying neighborhood characteristics considered.

**Meaning:**

These findings suggest that factors beyond neighborhood deprivation may contribute to increased breast cancer mortality among Black women.

## Teaser

This cohort study investigates whether neighborhood deprivation is associated with breast cancer mortality among Black and White women and whether rurality, residential mobility, and racial composition of the neighborhood modify that association.

## Introduction

Breast cancer is the second leading cause of cancer death among US women.^1^ Black US women, who are disproportionately burdened by breast cancer mortality, are 40% more likely than White women to die from the disease.^1^ The Black-White disparity is even higher in some states, including Georgia, where breast cancer is the leading cause of cancer death among Black but not White women.^2^ Many factors contribute to this disparity, including differential access to quality treatment, high prevalence of comorbidities, and unfavorable tumor characteristics.^3^ Despite this knowledge and recent declines in mortality rates, the breast cancer mortality racial disparity persists.^1^

Emerging evidence suggests that racial differences in social determinants of health contribute to breast cancer disparities.^4,5^ The neighborhood environment is one social determinant that is racially patterned. In the US, Black individuals are 4 times more likely than White individuals to live in deprived, low socioeconomic neighborhoods,^6^ which lack physical, economic, and social resources. Living in deprived neighborhoods may impact health and breast cancer mortality by increasing exposure to chronic stress,^7,8^ reducing health care access, and limiting opportunities for healthy behaviors.^8^ Thus, neighborhood deprivation, or low neighborhood socioeconomic status (SES), is hypothesized as a potential contributor to the breast cancer mortality racial disparity.^7^ Overall, studies demonstrate that neighborhood deprivation is associated with increased breast cancer mortality,^9,10,11^ particularly among White women.^11,12,13,14,15^ Literature examining the association among Black women is limited and has provided inconsistent results, with positive,^11,12,13^ inverse,^15^ and null^14,16^ associations reported, suggesting that further interrogation is needed. Prior research indicates accounting for multiple neighborhood characteristics and their interaction with neighborhood deprivation may elucidate the association between neighborhood and breast cancer mortality across race and ethnicity.^17^ Taking a similar robust approach may be necessary for enhancing our understanding of the association between neighborhood deprivation and breast cancer mortality among Black women, because interacting neighborhood factors may exacerbate or buffer the harmful impact of neighborhood deprivation. However, to our knowledge, no study has examined joint interactions of race and multiple neighborhood characteristics with the association.

In this study, we investigated the association between neighborhood deprivation and breast cancer mortality among Black and White patients with breast cancer in Georgia. To improve our understanding of the relationship among Black women, we assessed the association according to joint interactions of race and 3 neighborhood characteristics that may exacerbate or buffer neighborhood deprivation: rurality, neighborhood residential mobility, and neighborhood racial composition. Rurality may impact access to health care and resources^18^; decreased neighborhood residential mobility, a possible marker of social cohesion, may promote resilience^19^ and reduce inflammation^20^; and neighborhood racial composition, a marker of residential racial segregation, may influence experiences of racism.^21^ Examining the association according to joint interactions of race and these 3 neighborhood characteristics may help identify subgroups of women who are more susceptible to the adverse impact of neighborhood deprivation.

## Methods

### Study Population

In this cohort study, non-Hispanic Black and non-Hispanic White women identified by the Georgia Cancer Registry (GCR), a state-wide population-based cancer registry, as receiving a breast cancer diagnosis in 2010 to 2017 were eligible for study inclusion (53 264 women). Women younger than 18 years at diagnosis (3 women), with an autopsy or death certificate only diagnosis (238 women), incomplete dates or zero follow-up (103 women), in situ breast cancer (9737 women), stage IIIB to IV disease (4824 women), or missing data on breast cancer stage (991 women), block group (25 women), or neighborhood deprivation (548 women) were excluded. This cohort study followed the Strengthening the Reporting of Observational Studies in Epidemiology (STROBE) reporting guidelines. The Emory University institutional review board approved this study and granted a waiver of informed consent because of the use of deidentified GCR data and the nature of the study, in accordance with 45 CFR §46.

### Exposure and Outcome Ascertainment

Neighborhood deprivation was measured via the Neighborhood Deprivation Index (NDI), created by Messer and colleagues.^22^ The NDI comprises 8 indicators from the 2011 to 2015 American Community Survey (ACS): percentage of individuals with annual incomes below the federal poverty line, percentage of households receiving public assistance, percentage of female-headed households with children younger than 18 years, percentage of households with annual income less than $35 000, percentage of unemployed individuals, percentage of individuals employed in managerial or administrative jobs, percentage of households with more than 1 person per room, and percentage of individuals aged 25 years or older without a high school degree or General Educational Development credentials. Each indicator was measured at the US Census block group level, which we considered as the best approximation of a neighborhood, for all Georgia block groups. Principal component analysis was used to derive the NDI. We retained the first principal component, used loading values to weight each standardized indicator, and summed indicators to create an NDI score. Scores were categorized into quintiles on the basis of the Georgia distribution, such that the highest quintile represented the most deprived neighborhoods. The NDI was linked to patients’ addresses at diagnosis, which was collected and geocoded by the GCR. Vital status and cause of death, including breast cancer–specific death, was identified by the GCR through linkage to the Georgia vital statistics registry and National Death Index.

### Statistical Analysis

Data were analyzed between January 2023 and October 2023. We calculated descriptive statistics for covariates overall and across NDI quintiles. Cox proportional hazards regression was used to estimate age-adjusted and multivariable-adjusted hazard ratios (HRs) and 95% CIs for the association between neighborhood deprivation and breast cancer mortality. Participants accrued person-time beginning at breast cancer diagnosis and ending at breast cancer mortality, death from another cause, date of last follow-up, or end of the study period on December 31, 2022, whichever occurred first. Age (continuous), race (non-Hispanic Black or non-Hispanic White), marital status (single, married or living together, divorced or separated, widowed, and unknown), and county-level rurality (urban or rural) were identified as confounders on the basis of prior literature and graphical methods^23^ and were included as covariates in multivariable-adjusted models. All covariates were measured by the GCR at diagnosis, except rurality, which was obtained from the Georgia Department of Public Health. Rural counties were defined as having a population of less than 50 000 on the 2010 decennial US Census.

We conducted 3 sensitivity analyses. In the first, we estimated the association between neighborhood deprivation and breast cancer mortality using generalized estimating equations to account for potential clustering among Georgia neighborhoods. Estimates were similar to primary results. Thus, we present results from the primary analysis using Cox proportional hazards regression. In the second sensitivity analysis, we investigated potential exposure misclassification by examining the association between neighborhood deprivation and breast cancer mortality using 2 alternative deprivation indices: the Area Deprivation Index (ADI),^24^ which is available through the Neighborhood Atlas,^25^ and the Yost index, which is a neighborhood SES measure developed by Yost and colleagues.^26^ Derivation of the ADI and Yost index has been previously described.^24,26^ In short, principal component analysis of 2011 to 2015 ACS data on 17 and 7 indicators measured at the block group level was used to create the ADI and Yost index, respectively. Comparison of the NDI, ADI, and Yost index in a prior study^27^ showed the 3 deprivation indices to be similarly associated with breast cancer mortality. Analogously, we found nearly identical results in the sensitivity and primary analyses, and, therefore, only present the primary analysis. Results for the ADI and Yost index are presented in eTable 1 in Supplement 1. In the third, we conducted a competing risks analysis using Fine and Gray regression models. Estimates were similar to the primary analysis and thus, are not presented.

Because of the persistent Black-White racial disparity in breast cancer mortality,^1^ we examined race (non-Hispanic Black and non-Hispanic White) as an effect measure modifier of the neighborhood deprivation-breast cancer mortality association. We also investigated effect modification by rurality (urban vs rural), neighborhood residential mobility (less than or equal to the median value of 13.6% vs greater than the median value), and neighborhood racial composition (less than or equal to the median value of 19.0% vs greater than the median). Neighborhood residential mobility (ie, the percentage of residents who moved into or out of the neighborhood in the past year) and neighborhood racial composition (ie, the percentage of Black residents in the neighborhood) were measured using block group–level 2011 to 2015 ACS data. We performed additional analyses stratifying jointly on race and rurality, neighborhood residential mobility, or neighborhood racial composition. In these analyses, we used a common referent approach, such that within strata of race, the lowest quintile of neighborhood deprivation along with urban neighborhoods, neighborhoods with less than or equal to the median percentage of residential mobility, or neighborhoods with less than or equal to the median percentage of Black residents were considered the reference groups. Multiplicative interaction was assessed using likelihood ratio tests. *P* values for interaction were 2-sided with a .05 significance level. In a secondary analysis, we separately examined each of the 8 individual neighborhood deprivation indicators in relation to breast cancer mortality to better understand which NDI components may be underlying the observed associations. We performed analyses using SAS statistical software version 9.4 (SAS Institute).

## Results

Among the 36 795 participants included in the analytic sample (mean [SD] age at diagnosis, 60.3 [13.1] years), 11 044 (30.0%) women were non-Hispanic Black, 25 751 (70.0%) were non-Hispanic White, and 18 903 (51.4%) had private health insurance. We identified 20 006 women (54.4%) with stage I and 24 901 (67.7%) with luminal A breast cancer. Compared with women in the lowest quintile of NDI, those living in the highest quintile were more likely to be non-Hispanic Black, single, have Medicaid insurance, have triple-negative breast cancer, and live in a rural area with persistent poverty, high mobility, and a larger percentage of Black residents (Table 1). Race-specific characteristics are presented in eTable 2 in Supplement 1.

**Table 1.** Characteristics of the Study Population Overall and According to Quintiles of the NDI

Characteristics	Participants, No. (%)					
	Overall (N = 36 795)	NDI quintile^a^				
		First (least deprived) (n = 10 354)	Second (n = 8815)	Third (n = 7288)	Fourth (n = 5996)	Fifth (most deprived) (n = 4342)
Patient characteristics						
Age at diagnosis, mean (SD), y	60.3 (13.1)	59.7 (12.9)	59.9 (13.0)	60.8 (13.1)	60.6 (13.3)	61.0 (13.1)
Survival duration, median (IQR), mo	92.5 (50.8)	95.1 (50.7)	93.5 (49.2)	90.6 (50.6)	90.7 (52.6)	88.2 (51.8)
Race and ethnicity						
Non-Hispanic Black	11 044 (30.0)	1312 (12.7)	2240 (25.4)	2286 (31.4)	2527 (42.1)	2679 (61.7)
Non-Hispanic White	25 751 (70.0)	9042 (87.3)	6575 (74.6)	5002 (68.6)	3469 (57.9)	1663 (38.3)
Marital status						
Single	5038 (13.7)	980 (9.5)	1035 (11.7)	989 (13.6)	1005 (16.8)	1029 (23.7)
Married or living together	20 089 (54.6)	6943 (67.1)	5125 (58.1)	3749 (51.4)	2751 (45.9)	1521 (35.0)
Divorced or separated	4969 (13.5)	1070 (10.3)	1119 (12.7)	1034 (14.2)	952 (15.9)	794 (18.3)
Widowed	5126 (13.9)	1081 (10.4)	1159 (13.1)	1167 (16.0)	973 (16.2)	746 (17.2)
Unknown	1573 (4.3)	280 (2.7)	377 (4.3)	349 (4.8)	315 (5.2)	252 (5.8)
Insurance type						
Uninsured	589 (1.6)	105 (1.0)	151 (1.7)	123 (1.7)	102 (1.7)	108 (2.5)
Private	18 903 (51.4)	6272 (60.6)	4749 (53.9)	3464 (47.5)	2773 (46.2)	1645 (37.9)
Medicaid	2522 (6.8)	277 (2.7)	456 (5.2)	558 (7.7)	584 (9.7)	647 (14.9)
Medicare	13 417 (36.5)	3370 (32.5)	3077 (34.9)	2851 (39.1)	2325 (38.8)	1794 (41.3)
Military	795 (2.2)	211 (2.0)	242 (2.7)	172 (2.4)	111 (1.8)	59 (1.4)
Other or unknown	569 (1.5)	119 (1.1)	140 (1.6)	120 (1.6)	101 (1.7)	89 (2.0)
Cancer stage						
I	20 006 (54.4)	6099 (58.9)	4823 (54.7)	3837 (52.6)	3125 (52.1)	2122 (48.9)
II	14 126 (38.4)	3600 (34.8)	3373 (38.3)	2900 (39.8)	2416 (40.3)	1837 (42.3)
IIIA	2663 (7.2)	655 (6.3)	619 (7.0)	551 (7.6)	455 (7.6)	383 (8.8)
Molecular subtype						
Luminal A (HR^+^/ERBB2^−^)	24 901 (67.7)	7361 (71.1)	6006 (68.1)	4869 (66.8)	3930 (65.5)	2735 (63.0)
Luminal B (HR^+^/ERRBB2^+^)	4097 (11.1)	1133 (10.9)	990 (11.2)	831 (11.4)	681 (11.4)	462 (10.6)
HER2 overexpressing (HR^−^/ERBB2^+^)	1455 (3.9)	363 (3.5)	339 (3.8)	292 (4.0)	260 (4.3)	201 (4.6)
Triple negative (HR^−^/ERBB2^−^)	4370 (11.9)	939 (9.1)	1002 (11.4)	914 (12.5)	804 (13.4)	711 (16.4)
Unknown	1972 (5.4)	558 (5.4)	478 (5.4)	382 (5.2)	321 (5.3)	233 (5.4)
Neighborhood characteristics						
Rural	8702 (23.6)	776 (7.5)	1831 (20.8)	2406 (33.0)	2325 (38.8)	1364 (31.4)
Percentage living in persistent poverty^b^	3449 (9.4)	61 (0.59)	172 (1.95)	447 (6.13)	971 (16.19)	1798 (41.41)
Percentage of individuals moved in past year greater than the median^c^	18 296 (49.7)	4000 (38.63)	3903 (44.3)	3705 (50.8)	3526 (58.8)	3162 (72.8)
Percentage of Black residents greater than the median^d^	18 302 (49.7)	2166 (20.92)	4154 (47.1)	3989 (54.7)	4262 (71.1)	3731 (85.9)

Abbreviations: HR, hormone receptor; NDI, Neighborhood Deprivation Index.

^a^ Median NDI values per quintile are −2.32 for quintile 1, −1.11 for quintile 2, −0.20 for quintile 3, 0.88 for quintile 4, and 2.50 for quintile 5.

^b^ Persistent poverty is defined as US Census tracts with 20% or more of the population identified as living below the federal poverty level on the 1990 and 2000 decennial Censuses and in the 2007 to 2011 and 2015 to 2019 American Community Survey 5-year estimates.

^c^ Median neighborhood residential mobility is 13.6%.

^d^ Median percentage of Black residents in a neighborhood is 19%.

During follow-up, 2942 women (1214 [41.3%] non-Hispanic Black women and 1728 [58.7%] non-Hispanic White women) died from breast cancer. In age-adjusted models, neighborhood deprivation was associated with an increase in breast cancer mortality (NDI quintile 5 vs 1, HR, 1.90; 95% CI, 1.69-2.14) (Table 2). Additional adjustment for race decreased the HR (quintile 5 vs 1, HR, 1.49; 95% CI, 1.31-1.70). In multivariable models additionally adjusting for marital status and rurality, effect estimates were further reduced but persisted (quintile 5 vs 1, HR, 1.36; 95% CI, 1.19-1.55).

**Table 2.** Age-Adjusted and Multivariable-Adjusted HRs for the Association Between the NDI and Breast Cancer Mortality

NDI quintile	Breast cancer deaths, No.	Person-months, No.	HR (95% CI)		
			Age adjusted	Age and race adjusted	Multivariable adjusted^a^
First (least deprived)	623	988 080	1.00 [Reference]	1.00 [Reference]	1.00 [Reference]
Second	646	822 326	1.24 (1.11-1.39)	1.16 (1.04-1.30)	1.12 (1.00-1.26)
Third	623	660 788	1.48 (1.32-1.65)	1.34 (1.20-1.50)	1.26 (1.12-1.42)
Fourth	585	543 081	1.69 (1.51-1.90)	1.46 (1.30-1.64)	1.35 (1.20-1.52)
Fifth (most deprived)	465	383 748	1.90 (1.69-2.14)	1.49 (1.31-1.70)	1.36 (1.19-1.55)

Abbreviations: HR, hazard ratio; NDI, Neighborhood Deprivation Index.

^a^ HRs were also adjusted for rurality and marital status.

Stratified analyses revealed racial differences in the association between neighborhood deprivation and breast cancer mortality. Among non-Hispanic White women, living in the highest NDI quintile was associated with an increase in breast cancer mortality (quintile 5 vs 1, HR, 1.47; 95% CI, 1.21-1.79) (Table 3). No association was observed among non-Hispanic Black women, although the estimate for quintile 5 vs 1 was above 1 (HR, 1.12; 95% CI, 0.91-1.38). Rurality, neighborhood residential mobility, and neighborhood racial composition did not modify the association between neighborhood deprivation and breast cancer mortality.

**Table 3.** Age-Adjusted and Multivariable-Adjusted HRs for the Association Between the NDI and Breast Cancer Mortality According to Race, Rurality, Neighborhood Residential Mobility, and Neighborhood Racial Composition

Stratifying variable and NDI quintile	Breast cancer deaths, No.	Person-months, No.	aHR (95% CI)		Breast cancer deaths, No.	Person-months, No.	aHR (95% CI)	
			Age	Multivariable^a^			Age	Multivariable^a^
Race^b^	Non-Hispanic White				Non-Hispanic Black			
First (least)	487	869 633	1.00 [Reference]	1.00 [Reference]	136	118 448	1.00 [Reference]	1.00 [Reference]
Second	438	614 643	1.25 (1.10-1.43)	1.21 (1.06-1.38)	208	207 683	0.87 (0.70-1.08)	0.86 (0.69-1.07)
Third	380	455 820	1.43 (1.25-1.64)	1.35 (1.17-1.55)	243	204 969	1.03 (0.84-1.27)	1.00 (0.81-1.23)
Fourth	283	314 212	1.56 (1.34-1.80)	1.44 (1.24-1.69)	302	228 869	1.15 (0.94-1.41)	1.09 (0.89-1.34)
Fifth (most)	140	146 874	1.61 (1.34-1.95)	1.47 (1.21-1.79)	325	236 874	1.19 (0.98-1.46)	1.12 (0.91-1.38)
Rurality^c^	Urban counties				Rural counties			
First (least)	571	916 228	1.00 [Reference]	1.00 [Reference]	52	71 852	1.00 [Reference]	1.00 [Reference]
Second	503	654 994	1.23 (1.09-1.39)	1.11 (0.99-1.26)	143	167 332	1.18 (0.86-1.62)	1.12 (0.82-1.54)
Third	428	445 817	1.53 (1.35-1.73)	1.29 (1.13-1.47)	195	214 971	1.25 (0.92-1.70)	1.14 (0.84-1.55)
Fourth	367	335 164	1.75 (1.54-2.00)	1.37 (1.19-1.58)	218	207 917	1.45 (1.07-1.96)	1.25 (0.92-1.69)
Fifth (most)	324	265 062	1.94 (1.70-2.23)	1.39 (1.19-1.61)	141	118 686	1.63 (1.19-2.25)	1.25 (0.90-1.74)
Neighborhood residential mobility^d^	Less than or equal to the median percentage who moved^e^				Greater than the median percentage who moved			
First (least)	370	607 206	1.00 [Reference]	1.00 [Reference]	253	380 874	1.00 [Reference]	1.00 [Reference]
Second	348	459 941	1.24 (1.07-1.44)	1.12 (0.97-1.30)	298	362 386	1.23 (1.04-1.45)	1.12 (0.95-1.33)
Third	318	323 842	1.59 (1.37-1.84)	1.38 (1.18-1.62)	305	336 946	1.35 (1.14-1.59)	1.14 (0.96-1.35)
Fourth	235	225 978	1.70 (1.44-2.00)	1.40 (1.17-1.67)	350	317 103	1.64 (1.39-1.93)	1.29 (1.09-1.53)
Fifth (most)	111	104 945	1.72 (1.39-2.13)	1.29 (1.03-1.63)	354	278 803	1.88 (1.60-2.21)	1.33 (1.12-1.59)
Neighborhood racial composition^f^	Less than or equal to the median percentage of Black residents^g^				Greater than the median percentage of Black residents			
First (least)	470	784 745	1.00 [Reference]	1.00 [Reference]	153	203 335	1.00 [Reference]	1.00 [Reference]
Second	326	435 270	1.24 (1.07-1.42)	1.18 (1.02-1.36)	320	387 056	1.10 (0.90-1.33)	1.01 (0.84-1.23)
Third	264	299 330	1.44 (1.24-1.67)	1.32 (1.12-1.55)	359	361 458	1.31 (1.08-1.58)	1.15 (0.95-1.40)
Fourth	142	158 196	1.47 (1.22-1.77)	1.35 (1.10-1.64)	443	384 886	1.52 (1.26-1.82)	1.28 (1.05-1.54)
Fifth (most)	46	55 885	1.35 (1.00-1.82)	1.15 (0.85-1.57)	419	327 863	1.68 (1.39-2.02)	1.32 (1.08-1.60)

Abbreviations: HR, hazard ratio; NDI, Neighborhood Deprivation Index.

^a^ Multivariable HRs were adjusted for age at diagnosis, race, urban vs rural status, and marital status.

^b^ *P* for interaction = .05.

^c^ *P* for interaction = .86.

^d^ *P* for interaction = .44.

^e^ Median neighborhood residential mobility is 13.6%.

^f^ *P* for interaction = .43.

^g^ Median percentage of Black residents in a neighborhood is 19%.

In jointly stratified analyses, we observed similar race-specific patterns. For example, in analyses stratified jointly by race and rurality, neighborhood deprivation was associated with increased breast cancer mortality among non-Hispanic White women, regardless of rurality (NDI quintile 5 urban counties vs quintile 1 urban counties, HR, 1.39; 95% CI, 1.08-1.79; NDI quintile 5 rural counties vs quintile 1 urban counties, HR, 1.73; 95% CI, 1.34-2.24) (Table 4). Among non-Hispanic Black women, neighborhood deprivation was not significantly associated with breast cancer mortality across strata of rurality. However, effect estimates were above 1. In analyses examining the joint interactions of race and neighborhood residential mobility, as well as race and neighborhood racial composition, similar positive associations between neighborhood deprivation and breast cancer mortality were observed among non-Hispanic White women, regardless of the stratifying factor (Tables 4). Among non-Hispanic Black women, an HR greater than 1 was observed for those living in the most deprived high mobility neighborhoods, but the finding was not statistically significant (NDI quintile 5 greater than median mobility vs quintile 1 less than or equal to median mobility, HR, 1.28; 95% CI, 0.97-1.68). NDI was not associated with breast cancer mortality among non-Hispanic Black women, irrespective of neighborhood racial composition. Finally, of the 8 NDI components, the percentage of households receiving public assistance and household crowding had the highest HR estimates for breast cancer mortality (eTable 3 in Supplement 1).

**Table 4.** Multivariable-Adjusted HRs for the Association Between NDI and Breast Cancer Mortality According to the Joint Interactions of Race and Rurality, Neighborhood Residential Mobility, and Neighborhood Racial Composition

Stratifying variable and NDI quintile	Breast cancer deaths, No.	Multivariable HR (95% CI)^a^	Breast cancer deaths, No.	Multivariable aHR (95% CI)^a^	Breast cancer deaths, No.	Multivariable aHR (95% CI)^a^	Breast cancer deaths, No.	Multivariable aHR (95% CI)^a^
Joint interaction of race and rurality^b^	Non-Hispanic White patients				Non-Hispanic Black patients			
	Urban counties		Rural counties		Urban counties		Rural counties	
First (least deprived)	440	1.00 [Reference]	47	1.26 (0.93-1.70)	131	1.00 [Reference]	5	0.88 (0.36-2.15)
Second	322	1.21 (1.05-1.40)	116	1.36 (1.10-1.66)	181	0.83 (0.66-1.04)	27	1.22 (0.81-1.85)
Third	234	1.45 (1.23-1.70)	146	1.35 (1.12-1.63)	194	0.96 (0.77-1.19)	49	1.28 (0.92-1.78)
Fourth	143	1.50 (1.24-1.81)	140	1.53 (1.27-1.85)	224	1.08 (0.87-1.35)	78	1.23 (0.93-1.63)
Fifth (most deprived)	72	1.39 (1.08-1.79)	68	1.73 (1.34-2.24)	252	1.15 (0.93-1.43)	73	1.12 (0.84-1.50)
Joint interaction of race and neighborhood residential mobility^c^	Non-Hispanic White patients				Non-Hispanic Black patients			
	Less than or equal to the median percentage who moved		Greater than the median percentage who moved		Less than or equal to the median percentage who moved		Greater than the median percentage who moved	
First (least deprived)	305	1.00 [Reference]	182	0.99 (0.83-1.19)	65	1.00 [Reference]	71	1.19 (0.85-1.67)
Second	244	1.20 (1.02-1.43)	194	1.22 (1.02-1.46)	104	0.85 (0.62-1.16)	104	1.05 (0.77-1.43)
Third	211	1.43 (1.19-1.72)	169	1.27 (1.05-1.53)	107	1.15 (0.84-1.57)	136	1.04 (0.77-1.39)
Fourth	136	1.52 (1.23-1.89)	147	1.38 (1.13-1.69)	99	1.09 (0.79-1.50)	203	1.24 (0.94-1.64)
Fifth (most deprived)	44	1.41 (1.02-1.95)	96	1.50 (1.19-1.90)	67	1.04 (0.73-1.48)	258	1.28 (0.97-1.68)
Joint interaction of race and neighborhood racial composition^d^	Non-Hispanic White patients				Non-Hispanic Black patients			
	Less than or equal to the median percentage of Black residents		Greater than the median percentage of Black residents		Less than or equal to the median percentage of Black residents		Greater than the median percentage of Black residents	
First (least deprived)	406	1.00 [Reference]	81	1.03 (0.81-1.31)	64	1.00 [Reference]	72	1.01 (0.72-1.41)
Second	288	1.23 (1.05-1.43)	150	1.20 (0.99-1.45)	38	0.92 (0.62-1.38)	170	0.85 (0.64-1.14)
Third	232	1.38 (1.16-1.63)	148	1.33 (1.10-1.61)	32	1.04 (0.68-1.59)	211	0.99 (0.75-1.31)
Fourth	124	1.36 (1.11-1.68)	159	1.53 (1.27-1.84)	18	1.29 (0.76-2.19)	284	1.09 (0.83-1.43)
Fifth (most deprived)	40	1.32 (0.95-1.84)	100	1.55 (1.24-1.94)	6	0.60 (0.26-1.38)	319	1.15 (0.87-1.50)

Abbreviations: aHR, adjusted hazard ratio; HR, hazard ratio; NDI, Neighborhood Deprivation Index.

^a^ Multivariable HRs were adjusted for age at diagnosis and marital status.

^b^ *P* for interaction = .16.

^c^ *P* for interaction = .21.

^d^ *P* for interaction = .29.

## Discussion

In this prospective cohort study of non-Hispanic Black and non-Hispanic White patients with breast cancer, we found that living in the most deprived neighborhoods was associated with a 36% increase in breast cancer mortality. We observed effect measure modification of the association by race, but not rurality, neighborhood residential mobility, or neighborhood racial composition. Notably, neighborhood deprivation was associated with a 47% increased risk of breast cancer mortality among non-Hispanic White women, but not non-Hispanic Black women. In jointly stratified analyses, the association among non-Hispanic White women persisted across strata of rurality, neighborhood residential mobility, and neighborhood racial composition. These results highlight the impact of neighborhood on breast cancer mortality among non-Hispanic White women and suggest that further investigation is necessary among non-Hispanic Black women.

Similar to our findings, most prior studies have observed an association between neighborhood deprivation and breast cancer mortality. Specifically, neighborhood deprivation has been associated with a 10% to 76% increase in breast cancer mortality.^9,10,11,12,13,14,28,29,30,31,32,33,34,35^ However, no association after adjusting for covariates has also been reported.^36,37,38^ Literature examining the association between neighborhood deprivation and breast cancer mortality according to race is sparse, but growing. Our findings suggest that the impact of neighborhood deprivation on breast cancer mortality may vary by race, with non-Hispanic White women living in deprived neighborhoods experiencing an increased risk of breast cancer mortality, whereas their non-Hispanic Black counterparts do not. Similar results were found in a smaller previous study^14^ of Black and White patients with breast cancer diagnosed within health care systems in Atlanta, Georgia. In another study,^15^ neighborhood deprivation was associated with increased breast cancer mortality among White women, but a nonsignificant decrease was found among Black women. Nevertheless, other studies have reported no effect measure modification by race, with neighborhood deprivation increasing breast cancer mortality among both Black and White women,^11,12,13^ or having no impact in either group.^16^ The present study is, to our knowledge, the first attempt to uncover potential race-specific heterogeneity in the association between neighborhood deprivation and breast cancer mortality by investigating multiple stratifying factors representing access to health care, social cohesion, and residential racial segregation. Jointly stratified analyses revealed no modification of the association among non-Hispanic White women. The increased but not significant and nonmonotonic HR among non-Hispanic Black women living in high mobility neighborhoods indicates a potential target for further investigation using comprehensive assessments of social cohesion and support.

Research on the neighborhood environment has provided biological and behavioral mechanisms through which neighborhood deprivation may influence breast cancer mortality. Deprived neighborhoods are characterized by poor physical (eg, increased litter; dilapidated buildings; noise, light, and air pollution; and lack of green spaces, good schools, and nutritious food stores), socioeconomic (eg, concentration of individuals living in poverty with fewer years of education), and social (eg, increased crime and reduced safety and social cohesion) features.^8^ These characteristics create a complex interconnected web of stressors that promote chronic physical, emotional, and psychosocial stress. The body responds to stress by activating the fight or flight response through the hypothalamic pituitary adrenal axis.^8^ Normal infrequent activation of this response system is healthy. However, frequent and sustained activation can dysregulate physiologic processes in the body, leading to increased inflammation, reduced immunity, and epigenetic modifications,^8,39^ which may be key pathways in breast cancer progression.^7^ In addition, neighborhood deprivation could inhibit residents from engaging in outdoor physical activity, getting restful sleep, eating healthy foods, and having access to quality health care. Thus, neighborhood deprivation could impact breast cancer mortality directly by influencing the tumor microenvironment, as well as indirectly by reducing access to healthful behaviors and quality treatment.

Results from the current analysis suggest that these biological and behavioral pathways linking neighborhood and breast cancer mortality may be important for non-Hispanic White women regardless of the rurality, mobility, or racial composition of the neighborhood, but not non-Hispanic Black women, despite their increased exposure to neighborhood deprivation. Several factors may explain the lack of association among non-Hispanic Black women. First, Black individuals may experience diminishing returns as they achieve economic and social advancement. For instance, it is hypothesized that, compared with their White counterparts, as Black individuals move up the socioeconomic ladder and move into higher SES neighborhoods, where they are underrepresented, factors such as structural barriers, stress, discrimination, racism, and weaker social networks, hinder their ability to navigate, take advantage of, and benefit from their newly acquired resources (eg, improved access to education, wealth, and health care).^40^ As a result, for Black individuals, higher SES may afford less protection against illness and poor health outcomes, such as breast cancer mortality. In contrast, White individuals gain more from socioeconomic advancement, but lose more when those advancements are removed.^40^ A second potential explanation is John Henryism, a hypothesis that prolonged, intensive active coping leads to detrimental health impacts.^41^ Black individuals in high SES neighborhoods may have increased exposure to psychosocial stressors, such as racism and discrimination, that require persistent active coping. This coping style could increase vulnerability to poor health outcomes, especially if they live in environments lacking adequate social or faith-based resources.^42^ Finally, the exposure profile of Black women may differ from that of White women. For example, compared with White women, Black women may experience a greater number, higher prevalence, or more unique exposures—in addition to neighborhood deprivation—that influence breast cancer mortality, including structural racism, low individual-level SES, comorbidities, poorer tumor characteristics, and reduced access to quality health care.^3,43^

Although race modified the association between neighborhood deprivation and breast cancer mortality, rurality, neighborhood residential mobility, and racial composition did not. To our knowledge, few or no studies have investigated these 3 neighborhood factors as potential effect modifiers of the association. In 1 study^29^ of Hispanic women, low neighborhood SES was associated with increased breast cancer mortality, regardless of ethnic enclave level. Our results align with this prior study. However, more research is necessary.

### Limitations

There are limitations to the present analysis. Although our exposure, NDI, captures the multidimensionality of neighborhood deprivation, its interpretation cannot be easily translated into an intervention. However, assessment of the individual indicators used to create the NDI revealed that the percentage of households receiving public assistance and household crowding were most associated with breast cancer mortality (eTable 3 in Supplement 1), suggesting potential targets for intervention. The NDI characterizes neighborhood socioeconomic conditions, but other neighborhood factors, beyond socioeconomics, may be relevant to breast cancer mortality, such as the built environment, food environment, crime, and access to health care facilities. We measured NDI at a single time point, breast cancer diagnosis, which may not reflect length of residency, changes in address, or cumulative exposure to neighborhood deprivation over the life course. Nonetheless, individuals living in deprivation may be unlikely to move into socioeconomically different neighborhoods.^44^ Census block group was used to define neighborhoods, but these administrative boundaries may not coincide with individuals’ perceived neighborhood environment.^45^ Because this study used GCR data, which have limited individual-level data, we were unable to adjust for some individual-level confounders, including individual-level SES. In addition, our results may have limited generalizability to geographies beyond Georgia. However, similar findings have been reported across various geographic areas.^9,10,11,12,13,14,28,29,30,31,32,33,34,35^

## Conclusions

In this cohort study, we found that neighborhood deprivation was associated with increased breast cancer mortality among non-Hispanic White women but not non-Hispanic Black women. Further investigation of neighborhood residential mobility may help identify subgroups of non-Hispanic Black women at increased risk. However, other factors beyond those explored may contribute to increased breast cancer mortality among Black women and should be interrogated.

## Notes

Supplement 1. **eTable 1.** Age- and multivariable-adjusted hazard ratios for the association between neighborhood deprivation and breast cancer mortality **eTable 2.** Characteristics of the study population according to quintiles of neighborhood deprivation index and race, N=36,795 **eTable 3.** Age- and multivariable-adjusted models for the association between a 10-percentage point increase in the Neighborhood Deprivation Index component and breast cancer mortality

Supplement 2.
Data Sharing Statement
