# Supplementary material for: Neighborhood Deprivation and Breast Cancer Mortality Among Black and White Women
Source: JAMA Netw Open. 2024 Jun 12;7(6):e2416499. doi: 10.1001/jamanetworkopen.2024.16499 (PMC11170302; doi:10.1001/jamanetworkopen.2024.16499)
Supplement: Supplement 1. — eTable 1. Age- and multivariable-adjusted hazard ratios for the association between neighborhood deprivation and breast cancer mortality eTable 2. Characteristics of the study population according to quintiles of neighborhood deprivation index and race, N=36,795 eTable 3. Age- and multivariable-adjusted models for the association between a 10-percentage point increase in the Neighborhood Deprivation Index component and breast cancer mortality [file jamanetwopen-e2416499-s001.pdf]

Supplemental Online Content

Barber LE, Maliniak ML, Moubadder L, et al. Neighborhood deprivation and breast cancer mortality among Black and White women. *JAMA Netw Open*. 2024;7(6):e2416499. doi:10.1001/jamanetworkopen.2024.16499

eTable 1. Age- and multivariable-adjusted hazard ratios for the association between neighborhood deprivation and breast cancer mortality

eTable 2. Characteristics of the study population according to quintiles of neighborhood deprivation index and race, N=36,795

eTable 3. Age- and multivariable-adjusted models for the association between a 10-percentage point increase in the Neighborhood Deprivation Index component and breast cancer mortality

This supplemental material has been provided by the authors to give readers additional information about their work.

© 2024 Barber LE et al. *JAMA Network Open.*

eTable 1. Age- and multivariable-adjusted hazard ratios for the association between neighborhood deprivation and breast cancer mortality

	Breast cancer deaths	Person-months	Age-adjusted HR (95% CI)	Age and race-adjusted HR (95% CI)	MV-adjusted HR (95% CI) [a]
Area deprivation Index					
Q1 (Least deprived)	595	966,928	1.00 (Ref)	1.00 (Ref)	1.00 (Ref)
Q2	666	853,062	1.26 (1.13-1.41)	1.19 (1.06-1.33)	1.15 (1.03-1.29)
Q3	637	648,476	1.59 (1.42-1.78)	1.42 (1.27-1.59)	1.36 (1.21-1.52)
Q4	588	538,477	1.76 (1.57-1.97)	1.50 (1.34-1.69)	1.39 (1.23-1.58)
Q5 (Most deprived)	456	391,081	1.87 (1.65-2.11)	1.50 (1.32-1.70)	1.36 (1.19-1.57)
Yost Index					
Q5 (Least deprived)	596	967,029	1.00 (Ref)	1.00 (Ref)	1.00 (Ref)
Q4	680	857,549	1.30 (1.17-1.46)	1.20 (1.07-1.34)	1.18 (1.05-1.32)
Q3	620	664,685	1.52 (1.36-1.70)	1.38 (1.23-1.55)	1.32 (1.17-1.49)
Q2	589	520,648	1.83 (1.63-2.06)	1.61 (1.43-1.81)	1.52 (1.34-1.72)
Q1 (Most deprived)	457	388,112	1.89 (1.67-2.14)	1.53 (1.35-1.74)	1.42 (1.24-1.63)

HR, Hazard ratio; CI, Confidence interval; MV, Multivariable; Q, Quintile.
[a] Hazard ratios additionally adjusted for rurality and marital status.

© 2024 Barber LE et al. *JAMA Network Open.*

eTable 2. Characteristics of the study population according to quintiles of neighborhood deprivation index and race, N=36,795

	Neighborhood deprivation index									
	Non-Hispanic Black n= 11,044					Non-Hispanic White n= 25,751				
**Characteristics**	**Quintile 1 (Least) n=1,312**	**Quintile 2 n=2,240**	**Quintile 3 n=2,286**	**Quintile 4 n=2,527**	**Quintile 5 (Most) n=2,679**	**Quintile 1 (Least) n=9,042**	**Quintile 2 n=6,575**	**Quintile 3 n=5,002**	**Quintile 4 n=3,469**	**Quintile 5 (Most) n=1,663**
**Patient characteristics**	**Mean (SD)**	**Mean (SD)**	**Mean (SD)**	**Mean (SD)**	**Mean (SD)**	**Mean (SD)**	**Mean (SD)**	**Mean (SD)**	**Mean (SD)**	**Mean (SD)**
Age at diagnosis	55.3 (13.0)	55.9 (12.4)	56.9 (12.8)	58.1 (13.3)	59.3 (13.1)	60.4 (12.8)	61.3 (12.9)	62.6 (12.9)	62.4 (13.0)	63.6 (12.8)
Survival months	90.3 (35.5)	92.7 (34.1)	89.7 (36.2)	90.6 (36.6)	88.4 (37.4)	96.2 (33.9)	93.5 (35.0)	91.1 (35.9)	90.6 (36.7)	88.3 (37.6)
	**%**	**%**	**%**	**%**	**%**	**%**	**%**	**%**	**%**	**%**
Marital status										
Single	21.88	22.32	26.33	28.53	31.73	7.66	8.14	7.74	8.19	10.76
Married/living together	50.84	46.74	39.50	34.47	27.44	69.41	62.02	56.90	54.19	47.26
Divorced/separated	14.71	16.12	16.75	17.89	19.26	9.70	11.53	13.01	14.41	16.72
Widowed	8.38	10.22	12.25	13.45	15.34	10.74	14.14	17.73	18.25	20.14
Unknown	4.19	4.60	5.16	5.66	6.23	2.49	4.17	4.62	4.96	5.11
Insurance type										
Uninsured	1.91	2.14	2.58	2.10	2.95	0.88	1.57	1.28	1.41	1.74
Private	63.11	59.69	52.10	49.23	38.97	60.21	51.89	45.44	44.08	36.14
Medicaid	6.94	7.99	12.51	13.93	18.37	2.06	4.21	5.44	6.69	9.32
Medicare	22.87	23.88	27.73	31.06	36.36	33.95	38.66	44.32	44.39	49.31
Military	4.27	4.82	3.54	1.98	1.64	1.71	2.04	1.82	1.76	0.90
Other/unknown	0.91	1.47	1.53	1.70	1.72	1.18	1.63	1.70	1.67	2.59
Stage										
I	46.34	46.12	45.84	46.42	44.23	60.73	57.64	55.76	56.27	56.34
II	45.12	45.31	44.01	44.32	45.13	33.27	35.86	37.86	37.36	37.76
IIIA	8.54	8.57	10.15	9.26	10.64	6.01	6.49	6.38	6.37	5.89
Molecular subtype										
Luminal A (HR+/HER2-)	60.44	58.97	59.23	58.61	58.23	72.64	71.25	70.27	70.60	70.66
Luminal B (HR+/HER2+)	11.59	12.37	12.03	12.39	11.09	10.85	10.84	11.12	10.61	9.92
HER2 overexpressing (HR-/HER2+)	4.95	5.36	4.68	5.58	5.23	3.30	3.33	3.70	3.43	3.67

© 2024 Barber LE et al. *JAMA Network Open.*

Triple negative (HR-/HER2-)	17.68	17.95	18.29	18.12	20.57	7.82	9.13	9.92	9.97	9.62
Unknown	5.34	5.36	5.77	5.30	4.89	5.40	5.44	5.00	5.39	6.13
**Neighborhood characteristics**										
Percent living in persistent poverty [a]	0.30	2.05	6.78	19.67	50.17	0.63	1.92	5.84	13.66	27.30
Urban/rural status										
Urban	95.58	90.63	83.60	75.94	76.45	92.06	75.35	59.40	50.50	55.92
Rural	4.42	9.38	16.40	24.06	23.55	7.94	24.65	40.60	49.50	44.08
Percent individuals moved in past year: >median [b]	47.79	45.04	58.27	65.49	76.63	37.30	44.02	47.44	53.93	66.69
Percent of Black residents: >median [c]	52.90	82.90	87.36	95.09	96.83	16.28	34.94	39.82	53.59	68.37

SD, Standard deviation; HR, Hormone receptor; HER2, Human epidermal growth factor receptor 2.

[a] Persistent poverty defined as census tracts with ≥20% of the population identified as living below the poverty line on the 1990 and 2000 decennial censuses and in the 2007-2011 and 2015-2019 American Community Survey 5-year estimates.

[b] Median neighborhood residential mobility = 13.6%.

[c] Median percentage of Black residents in a neighborhood = 19%.

© 2024 Barber LE et al. *JAMA Network Open.*

eTable 3. Age- and multivariable-adjusted models for the association between a 10-percentage point increase in the Neighborhood Deprivation Index component and breast cancer mortality

NDI component	Age-adjusted HR (95% CI)	MV-adjusted HR (95% CI) [a]
Percent individuals below poverty line	1.13 (1.11-1.16)	1.05 (1.03-1.08)
Percent household receiving public assistance	1.37 (1.22-1.53)	1.20 (1.06-1.35)
Percent female-headed households with children age <18 years	1.18 (1.14-1.23)	1.04 (1.00-1.09)
Percent household income <$35,000	1.12 (1.10-1.14)	1.06 (1.04-1.08)
Percent employed in managerial/administrative jobs	0.80 (0.76-0.85)	0.90 (0.86-0.96)
Percent housing crowding	1.36 (1.24-1.50)	1.20 (1.09-1.33)
Percent individuals unemployed	1.20 (1.14-1.26)	1.07 (1.01-1.12)
Percent individuals age >25 without a high school degree or GED	1.16 (1.12-1.20)	1.09 (1.05-1.13)

NDI, Neighborhood Deprivation Index; HR, Hazard ratio; CI, Confidence interval; MV, Multivariable; GED, General Educational Development.
[a] Multivariable hazard ratios adjusted for age at diagnosis, race, urban/rural status, and marital status.

© 2024 Barber LE et al. *JAMA Network Open.*
