# Supplementary material for: Neighborhood Deprivation and Breast Cancer Mortality Among Black and White Women
Source: JAMA Netw Open. 2024 Jun 12;7(6):e2416499. doi: 10.1001/jamanetworkopen.2024.16499 (PMC11170302; doi:10.1001/jamanetworkopen.2024.16499)
Supplement: Supplement 2. — Data Sharing Statement [file jamanetwopen-e2416499-s002.pdf]

Data Sharing Statement

Barber. Neighborhood Deprivation and Breast Cancer Mortality Among Black and White Women. *JAMA Netw Open*. Published June 12, 2024.
doi:10.1001/jamanetworkopen.2024.16499

Data
Data available: No

Additional Information
Explanation for why data not available: The datasets generated during and/or analyzed during the current study are not publicly available due to IRB protocol but are available from the corresponding author on reasonable request to the study PI (lauren.mccullough@emory.edu).
